# Using Pathway Covering to Explore Connections among Metabolites

**DOI:** 10.3390/metabo9050088

**Published:** 2019-05-02

**Authors:** Peter E. Midford, Mario Latendresse, Paul E. O’Maille, Peter D. Karp

**Affiliations:** 1 Bioinformatics Research Group, SRI, International, Menlo Park, CA 94025, USA; midford@ai.sri.com (P.E.M.); latendre@AI.SRI.COM (M.L.); 2 Biocomplexity Sciences, SRI International, Menlo Park, CA 94025, USA; paul.omaille@sri.com

**Keywords:** metabolite sets, set theory, optimization, pathways, BioCyc

## Abstract

Interpreting changes in metabolite abundance in response to experimental treatments or disease states remains a major challenge in metabolomics. Pathway Covering is a new algorithm that takes a list of metabolites (compounds) and determines a minimum-cost set of metabolic pathways in an organism that includes (covers) all the metabolites in the list. We used five functions for assigning costs to pathways, including assigning a constant for all pathways, which yields a solution with the smallest pathway count; two methods that penalize large pathways; one that prefers pathways based on the pathway’s assigned function, and one that loosely corresponds to metabolic flux. The pathway covering set computed by the algorithm can be displayed as a multi-pathway diagram (“pathway collage”) that highlights the covered metabolites. We investigated the pathway covering algorithm by using several datasets from the Metabolomics Workbench. The algorithm is best applied to a list of metabolites with significant statistics and fold-changes with a specified direction of change for each metabolite. The pathway covering algorithm is now available within the Pathway Tools software and BioCyc website.

## 1. Introduction

As interest in metabolomics grows, so does the number of data sets. For example, during the past three years (December 2015–2018), the available studies deposited in Metabolomics WorkBench [1] have increased from 161 to 816. Once a set of metabolites influenced by a treatment has been identified, a next step in understanding is interpreting the changes in the context of the organism’s complete metabolic network.

**Figure 1:**
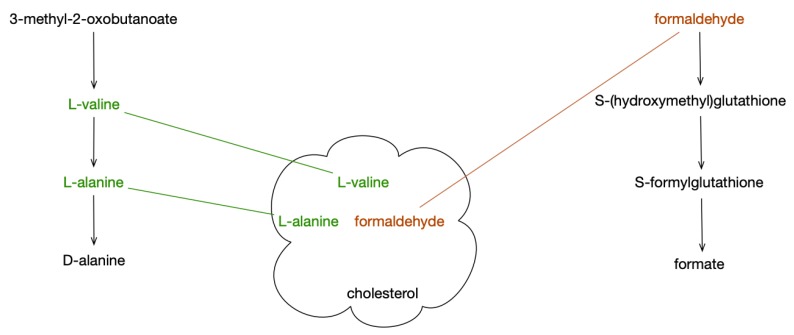
Schematic showing two pathways covering three compounds; the fourth compound has no pathways in the organism, and cannot be covered.

Pathway covering (Figure 1) is a new method for analyzing metabolite sets against a set of pathways. It is based on the set-cover problem [2] that has recently been applied to reducing redundancy among pathway databases [3], but it has not, to our knowledge, been used to suggest pathways associated with changes in metabolite levels. We implemented pathway covering as part of the Pathway Tools [4,5] software and evaluated it using the HumanCyc Pathway/Genome Database (PGDB) and several datasets from Metabolomics Workbench (MWB) and another published study. HumanCyc and Pathway Tools are components of the BioCyc [6] website and collection of more than 14,000 organism PGDBs.

The classic set-cover problem starts with a “universal” set of elements and a collection of sets of elements drawn from that universal set. The goal is identifying a smallest collection of these sets that covers the entire universal set of elements. One extension of this problem is to apply weights or costs to each of the sets that are combined to form a cover. This is called the weighted set-cover problem. We will refer to these weights as costs throughout this paper as our optimizer is designed to seek a minimum value solution.

In this work, the set to be covered consists of metabolites observed to have changed due to disease or experimental conditions, and the collection of sets meant to cover the input set of metabolites consists of the collection of sets of substrate compounds for each pathway in the organism. In this application, we assign costs to the pathways themselves, rather than their sets of substrate compounds.

The problem is finding a collection of sets (pathway substrates) that:

- together includes as many of the given input elements (metabolites) as possible and
- represents a “minimum cost” way of doing so.

Assigning a cost to each pathway and then solving for a minimum-cost solution reduces the possible solutions to sets of pathways that are small, thereby avoiding “solutions” such as the set of all pathways that contain one or more substrates on the list. Different analysis goals will suggest different cost functions that will yield alternative solutions. Such goals might include the absolute smallest number of pathways, or pathways that are small or have a large a proportion of input substrates, or pathways with a particular function or class such as synthesis or degradation.

The problem of finding the smallest covering set is one of the well-known NP-hard problems [2], and several approaches to solving or estimating solutions have been developed. These approaches may provide approximate or exact optima (in terms of minimizing cost) and will use different types of algorithms. Two common solution approaches are “greedy” algorithms [7,8] (approximate solutions), and algorithms that transform the set-cover problem into an equivalent NP-complete problem (exact solutions), such as integer linear programming (ILP), for which efficient tools for finding solutions have been developed. Although ILP is potentially exponential, our experience is that for the sets of metabolites discussed here, the solver returns a solution in no more than one second on modern hardware (which is faster than the process of mapping input names to database objects which precedes the solver).

In practice, the only input metabolites that can be included in a covering solution are those that are recognized and are substrates of at least one pathway in the database. Many compounds in organism metabolic databases, such as HumanCyc, are not assigned to any metabolic pathway. In addition, pathways include compounds not found in the input set. These compounds can be removed from consideration as they have no bearing on whether a pathway will cover compounds in the input set. By extension, any pathway with no compounds in the input set is eliminated from further consideration.

Therefore, the pathway covering problem is to cover the subset of input metabolites that are known to be substrates of at least one pathway, using the subsets of the substrate compounds of pathways that occur in the input set. The optimal solution, at this point, is the smallest set of pathways that cover the compounds in the input set.

This approach will always yield the smallest collection of pathways or, in some cases several collections of pathways of equal size. However, this solution may not be the most biologically meaningful. For example, using the constant cost function will prefer larger pathways that include more input compounds, since fewer pathways will be needed for the cover. These larger pathways are more likely to have branches and alternative routes such that a particular compound may not be essential to the pathway, whereas a smaller pathway is less likely to have alternative routes. There may also be prior justification to focus on pathways of a certain type (e.g., biosynthetic pathways).

We explored more than a dozen cost functions, but we will report results for the five functions (enumerated below) that we are making available in the released version of the tool.

## 2. Results

### 2.1. Selection of Studies

The behavior of the pathway covering algorithm and a set of five cost functions were evaluated by using the following three studies.

- “Metabolic Profiling of Visceral and Subcutaneous Adipose Tissue from Colorectal Cancer Patients: GC-TOF MS analysis of subcutaneous and visceral adipose tissue samples” (Metabolomics Workbench (MWB) repository study ST000061; [9]). This study compares the metabolism of subcutaneous and visceral fat.
- “Metabolite-Phenotype Link in X-Linked Adrenoleukodystrophy (Fibroblast Cell Culture)” (MWB study ST000741; [10]). This study was an untargeted comparison of fibroblasts from subjects with either advanced stage or mild adrenoleukodystrophy disease against controls. The analysis presented here compared metabolites from cells collected from either control subjects or those subjects with an advanced disease state.
- “Integrated phosphoproteomic and metabolomic profiling reveals NPM-ALK-mediated phosphorylation of PKM2 and metabolic reprogramming in anaplastic large call lymphoma” [11] (data from an earlier stage of this study was deposited as Metabolomics Workbench study ST000016). For our analysis we used the set of metabolites listed in Table S2 of the Supplemental Material, available at [12].

### 2.2. Evaluating Solutions from Different Pathway Cost Functions

Our primary results consist of a pathway covering analysis of the three selected studies using five different cost functions. A more detailed description of the cost functions appears in the methods, but in summary, the cost functions are:

- **Constant**—This function returns a constant 1.0 for every pathway. This cost function yields solutions containing the smallest sets of pathways. This corresponds to the original set covering formulation of the problem.
- **Pathway Size**—This function returns the number of reactions in the pathway. This function shows a preference for small pathways over large ones. In particular, for pathway databases, such as HumanCyc, that include pathways nested within larger superpathways, this cost function will favor the smaller pathway if the superpathway does not cover more input compounds.
- **Biosynthesis-Preferred**—This function starts with the pathway size cost, then, if unless the pathway is a biosynthesis pathway, the cost is approximately doubled. The ontology of pathways in the HumanCyc PGDB is used to determine whether a pathway is biosynthetic or not.
- **Covered Compound Sparseness**—This function collects the substrate compounds for all reactions in the pathway. It divides this set between those compounds that are in the set of input compounds and the remainder. It divides the size of the remainder set by the size of the set of compounds in the input set.
- **Pathway harmony**—This function is designed to capture some of the features of pathway flux. It divides the substrate compounds of each pathway into three groups: inputs, outputs, and intermediates. For datasets for which direction of change is specified as increasing or decreasing, this function looks at whether substrates within each group are changing in the same direction (either all substrates in the group or a majority). Agreement within input or output covered metabolites are scored more highly than agreement within intermediates, and complete agreement is scored more highly than majority agreement. This function does not compare change directions between groups.

### 2.3. Selection of Compounds

From each study we extracted lists of significantly changed compounds. In two of the studies (ST000741, McDonnell et al.) we extracted separate lists of compounds that increased and decreased concentrations between the conditions compared in the study. The third study (ST000061) compared two cell lines and all significant concentration changes were in one line. Table 1 summarizes the input compounds from each study. The first column identifies the study. The second column counts the number of significant compounds selected for each study and the direction of change when appropriate. In the remaining study, all significant changes were in the same direction. The third column counts the number of study compounds that were found in the HumanCyc database. The fourth column lists the number of compounds that are included in at least one pathway that specifically listed the compound. Pathways in which the compound was only present as an instance of a class in a generic reaction (e.g., reactions involving a substrate “any amino acid”) were not considered.

Several compounds required manual resolution as the name provided by Metabolomics Workbench could not be automatically resolved. In the course of manual resolution, we identified several compounds that were found in the more extensive MetaCyc database from which HumanCyc was partially derived.

In some cases, an input compound name was ambiguous; it either referred to more than one compound or more commonly, it referred to what HumanCyc considered to be a class of compounds. For example, “Glucose” refers to a compound class that ultimately has three compound instances (leaves of the ontology tree), of which two (alpha-d-glucopyranose and beta-d-glucopyranose) have reactions that occur in pathways and need to be considered. Rather than trying to resolve the ambiguity, we included all possible interpretations.

### 2.4. Study ST000061—Visceral and Subcutaneous Adipose Tissue

This study compared two types of adipose tissue (subcutaneous and visceral) from 50 colo-rectal cancer patients, though 59 samples are reported for both cell types. All significant changes were in the direction of higher metabolite levels in the visceral tissue. There were 39 named compounds (Table 2) that showed a fold-change difference of 1.5 or greater and that also had a corrected *p*-value less than 0.1. These 39 named compounds were resolved to 45 metabolites known to HumanCyc. Of these 45, 35 were substrates in one or more pathways. Because all the changes were in the same direction, the results from the pathway harmony cost function were omitted, as these were not meaningful in this case. The details of solutions for each cost function are listed in Table A1 through Table A4.

Table 3 summarizes the solution sets returned by each cost function. The second column shows the number of solutions returned for each cost function, while the third column shows the size range of the solution sets. Finally, column four indicates the largest number of compounds that were covered by a pathway in the solution set.

The constant cost function generated over 1 million solution sets, and apart from a representative solution of 20 pathways listed in Table A1 in Appendix A we did not explore these solutions further. Because the solver objective function is equal to the size of the solution for this cost function, all optimal solutions will be the same size. The smallest number of solutions was returned by covered compound sparseness, which also returned solutions that were smaller in size than the other cost functions. Hence we will prefer this cost function for future analyses.

Of the 35 metabolites resolved from the 39 names reported in the study, 12 cases had a metabolite that was consistently covered by a particular pathway, across all solutions returned by any cost function.

- alpha-tocopherol covered by alpha-tocopherol degradation
- arachidonic acid and ethanolamine covered by anandamide degradation
- cholesterol covered by pregnenolone biosynthesis
- L-isoleucine covered by isoleucine degradation
- L-leucine covered by leucine degradation
- L-tyrosine and L-phenylalanine covered by tyrosine biosynthesis
- L-threonine covered by threonine degradation
- L-valine covered by valine degradation
- putrescine and L-ornithine covered by putrescine biosynthesis III

For eight compounds (alpha-tocopherol, arachidonic acid, ethanolamine, isoleucine, leucine, phenylalanine, threonine, and valine), the pathway selected was the only one in the database that specified the particular compound (as opposed to a class of compounds) as a substrate. Likewise, the presence of phenylalanine pulled in its degradation pathway which removed any advantage to including tyrosine in any other pathway. The absence of synthesis pathways is consistent with six of the seven listed amino acids being considered essential in humans. Likewise, alpha-tocopherol is a nutrient that humans cannot synthesize (vitamin E). Although both arachidonic acid and ethanolamine are specific substrates of multiple reactions in pathways, anandamide degradation is the only pathway in which they both occur.

The small solution set size returned by the compound sparseness cost function may reflect the function consistently covering seven compounds with one superpathway (“superpathway of conversion of glucose to acetyl CoA and entry into the TCA cycle”). The constant cost function also did this for at least some solutions. The remaining cost functions, size, and biosynthesis preferred, covered four of these seven with a smaller pathway (“Rapoport-Luebering glycolytic shunt”).

However, these four compounds were all introduced to the covering procedure by one input name (“glyceric acid”) which in HumanCyc is a class with four instance compounds. The glycolytic shunt is active in mammalian erythrocytes but is not reported to be active in adipose tissue.

Intuitively, if the input metabolite set represents a coordinated shift in metabolism as a result of the experiment, the size of its pathway covering set should be smaller than the pathway covering set for a set of metabolites of the same size, drawn at random from pathways in the organism. This notion was tested by generating 1000 sets of 34 compounds, drawn without replacement from all compounds that were substrates of at least one pathway. The covering algorithm was applied to each set, using the constant value cost function, and the size (number of pathways) in each solution was collected into a size distribution. Comparing the size *S* of the covering set returned for the study data (20) against the distribution, *S* was smaller than 994 coverings and equal in size to three coverings of random compound sets. This suggests that the pathway covering tool is finding a signal in the observed metabolite shifts that is absent in random collections of metabolites in pathways. As such it validates both the method and the dataset.

To compare pathway covering with enrichment, we performed a “compounds enriched for pathways” analysis of the 39 compounds used in the pathway covering analysis using HumanCyc as the database (see methods for details). A pathway enrichment analysis of the ST000061 data set (see Methods for details) returned ten individual pathways (and 13 pathway classes). We compared this result against the solutions returned by pathway covering using the covered compound sparseness function. The covering analysis returned four solutions, sharing 19 pathways with an additional five pathways appearing among alternative solutions (Appendix A Table A4), which we included. For this data set, there was relatively little overlap between the individual pathways from the enrichment and covering analyses (Table 4). Adding the five pathways that occurred in some covering solutions did not increase the overlap.

### 2.5. ST000741—X-Linked Adrenoleukodystrophy

This study compared metabolite levels in fibroblast cell cultures collected from a small number (*n* = 6 per condition) of patients with mild or severe ALD disease or healthy controls. The input list consists of extracted metabolites showing differences between the healthy and severe disease conditions. The compounds are listed in Table 5, and a summary of results appears in Table 6. The details of solutions for each cost function are listed in Table A5 through Table A9.

Of the 20 input metabolites, 12 cases had a metabolite that was consistently covered by a particular pathway, regardless of the cost function.

- docosahexaenoic acid covered by aspirin triggered resolvin D biosynthesis
- *n*-acetyl-beta-neuraminate covered by CMP-*N*-acetylneuraminate biosynthesis I (eukaryotes)
- N omega-phosphocreatine covered by creatine-phosphate biosynthesis
- nicotinamide covered by NAD salvage
- ophthalmate covered by ophthalmate biosynthesis
- sn-glycero-3-phosphocholine covered by plasmalogen degradation
- beta-d-fructofuranose and keto-d-fructofuranose covered by sorbitol degradation I
- beta-d-glucopyranose and alpha-d-glucopyranose covered by trehalose degradation
- alanine and taurine covered by tRNA-uridine 2-thiolation

For eight compounds (*N*-acetyl-beta-neuraminate, omega-phosphocreatine, nicotinamide, ophthalmate, sn-glycero-3-phosphocholine, choline, beta-d-fructofuranose, and keto-d-fructose), the pathway selected was the only one in the database that specified the particular compound. Likewise, the pairs beta-d-glucopyranose–alpha-d-glucopyranose and alanine–taurine were only found together in those particular pathways, though the first pair is the result of a disambiguation. This led to relatively few differences between cost functions. The constant and pathway harmony functions did pick the larger “superpathway of conversion of glucose to acetyl CoA and entry into the TCA cycle” over covering the same three compounds with the TCA cycle pathway. Since exactly the same three compounds were covered, this case illustrates the shortcoming of cost functions that ignored pathway size versus those that explicitly (pathway size, biosynthesis-preferred) or implicitly (covered compound sparseness) incorporated size.

As with ST000061, we compared the size of the covering set calculated with the constant cost function against the distribution of covering sets from 1000 sets of 20 metabolites generated at random from the metabolites with pathways in the HumanCyc database. The observed size of 14 pathways was smaller than 936 coverings and equal in size to 44 pathway coverings from the 1000 random sets. This finding is less significant than the result from ST000061, and suggests this set is possibly a lower-quality data set. This result is consistent with the fact that although all compounds in the input list showed significant and sizable changes, no compound changes in this data set were significant after correcting for false discovery rate [13].

A pathway enrichment analysis of the ST000741 compounds yielded the unexpected result that no pathways were returned when the Benjamini-Hochberg correction was applied, even with a cutoff *p*-value of 0.3. The analysis (Table 7) uses the 29 individual pathways returned from an enrichment with no multiple comparison correction and a cutoff *p*-value of 0.1. Nine of the 29 pathways were shared with the result from pathway covering using covered compound sparseness (which returned one solution). The later included six additional pathways. Unlike ST000061, this result shows that enrichment and pathway covering can substantially overlap.

### 2.6. McDonnell et al.—NPM-ALK Regulation

McDonnell et al. used a combination of phosphoproteomics and metabolomics to highlight changes in metabolism brought about by NPM-ALK. Their conclusions were derived from a range of methods. Here, we focus on the list of Metabolites in their Supplemental Table S2, which they claim are metabolites regulated by ALK. The table compares normalized metabolite abundance in control cells that were treated with DMSO and cells from the same line that were treated with an ALK inhibitor (CEP). No significance levels were reported for these metabolites, but fold-changes were, so, for consistency, we applied the same 1.5 fold-change cutoff as was done with the MWB studies. Figure 3 in their publication indicates that the list of metabolites may have been filtered at the *p* = 0.05 level. Table 8 lists the metabolites, while Table 9 summarizes the number of solutions for each cost function. The third column lists the range of solution sizes (number of pathways in the covering set) for each cost function. The fourth column lists the largest number of metabolites covered by a single pathway in any solution. The details of the solutions for each cost function are listed in Table A10 through Table A14.

Of the 19 metabolites resolved from the 24 names reported in the study, four metabolites were consistently covered to a particular pathway, regardless of the cost function.

- sn-glycero-3-phosphocholine covered by plasmalogen degradation
- lactate covered by pyruvate fermentation to (S)-lactate.
- palmitate covered by stearate biosynthesis
- AMP covered by stearate biosynthesis

Only one compound, sn-glycero-3-phosphocholine appeared in the database as a substrate only of its covering pathway. The inclusion of AMP, although appearing in many pathways, is the likely reason that palmitate (also covered by stearate biosynthesis) was never covered by its own synthesis pathway.

We compared the size of the covering set calculated with the constant cost function against the distribution of covering sets from 1000 sets of 19 metabolites generated at random from the metabolites with pathways in the HumanCyc database. The observed size of eight was smaller than any covering from a randomly generated set. This is not surprising since the list of compounds in the supplemental list was certainly not random.

McDonnell et al. [11] interpreted their metabolite results as showing changed activity for glycolysis, TCA cycle, and nucleotide metabolism. Our pathway covering results consistently included nucleotide metabolism and fatty acid synthesis. The constant cost function picked up glycolysis and the TCA cycle. Two others (pathway size and covered compound sparseness) picked up the glycerol-3-phosphate shuttle. Although McDonnell et al. identified NAD+ and succinate as characteristic of the TCA cycle, at least the biosynthesis-preferred cost function placed them together under L-carnitine biosynthesis. Thus, the TCA cycle is not the only pathway where they are found and these alternatives explain why TCA was not included by all cost functions.

Pathway enrichment of the McDonnell et al. data set returned 44 individual pathways that included nine of the ten pathways that the single solution from covered compound sparseness (Table 10). The enrichment results did show a tendency to report multiple pathways enriched for the same small sets of compounds, for example NAD+, coenzyme A, and AMP are the only enriched compounds in four pathways (three of which are variants of ethanol degradation). Since the same compounds are enriched in all four, the analysis provides no way to determine a preference. Since these compounds are “better“ covered elsewhere the pathway covering result includes none of them.

### 2.7. Pathway Covering Results Depicted in a Pathway Collage

**Figure 2:**
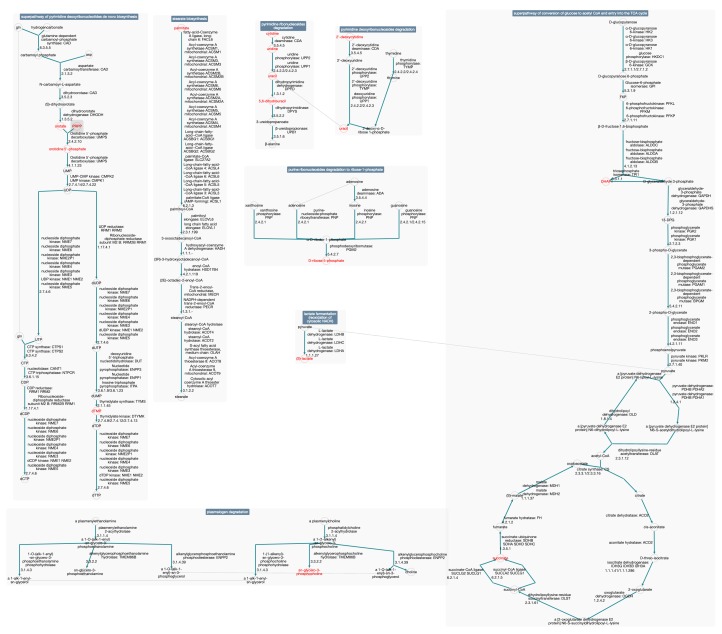
This is a pathway collage showing the pathways in the constant pathway cost solution for the McDonnell data set and highlighting most of the covered compounds. Three covered compounds (NAD+, coenzyme A, guanine) are not shown in the collage because they are considered side compounds of reactions (meaning they are not shared between consecutive reactions in the pathway) and are not drawn by the pathway layout algorithm. Two compounds, glycerone phosphate and 5-phospho-alpha-d-ribose 1-diphosphate are shown on the collage under different names, DHAP and PRPP.

Figure 2 shows a BioCyc “Pathway-Collage” [14] diagram showing a covering solution for the McDonnell data set using the constant cost function. The pathway-collage tool enables a user to create a multi-pathway diagram in which pathways are positioned relative to one another, connections among pathways can be displayed, and metabolites and genes can be highlighted. The BioCyc Pathway Covering tool can send a pathway covering solution directly to the Pathway-Collage tool.

## 3. Discussion

Any method that interprets metabolite measurements in terms of changes in pathways will necessarily be dependent on the database of pathways used for interpretation. Furthermore, the requirements that a compound is in the database, is recognized by name, and exists in at least one reaction and one pathway is common to all pathway-based interpretations of metabolomics data. The necessity to identify reactions and pathways led us to avoid studies with a large lipidomics component. Lipids pose a particular challenge since there are relatively few pathways known that are specific to particular lipid species even though they are easy to detect and identify with current methods. The solution lies in continued growth and curation of pathway databases, along with the caution that results with this method will differ between databases with different sets of metabolites and pathways.

There are several other approaches to the interpretation of metabolite lists in terms of affected pathways. A review by Booth et al. [15] divided the approaches at the time between visualization and enrichment (or over-representation)-based approaches. Although other, non-pathway-based approaches to interpreting metabolomics data (e.g., [16]), have been developed, the majority of pathway-based tools have used enrichment, which is extensively reviewed by Marco-Ramell et al. [17] and is also implemented in BioCyc [18]. Enrichment methods ask the question: is the input metabolite set over-represented for metabolites within certain pathways relative to what would be expected by chance, based on the complete set of pathways and metabolites (present in pathways).

The review by Marco-Ramell et al. covered 13 different tools. These included cross database tools such as MetaboAnalyst and its tools MSEA [19] and MetPA [20], as well as MBRole [21] and ConsensusPathDB [22], which draw pathway data from multiple databases, most commonly KEGG and HumanCyc as well as more specialized databases such as OMIM. Marco-Ramel et al. found fairly small differences among the tools, with potentially the biggest issue being tools that used out-of-date versions of the databases.

For each compound in the input list, enrichment methods require a determination of statistical significance. If no pathway achieves a usable *p*-value, either because of the pathway’s size or other properties of the database, nothing is reported. By contrast, the pathway covering algorithm assigns every compound to a pathway if such a pathway exists in the database, regardless of statistical significance. There will also be differences depending on the choice of statistical method (Hypergeometric/Fisher’s exact vs. Kolmogorov-Smirnov) and the correction for multiple comparisons (in most cases Benjamini-Hochberg [13]).

When we compared the results of enrichment and pathway covering empirically, we found that in one case (ST000061) pathway covering returned more pathways, and in two cases enrichment returned substantially more pathways. Our interpretation is that pathway covering will return more results when enrichment *p*-values do not reach statistical significance (e.g., in experiments with relatively few observed metabolites), which can be an advantage if in fact a single metabolite is a valid signal for a pathway whose flux is indeed changing. In particular, enrichment methods may tend to miss large pathways since to exceed the *p*-value cutoff many metabolites for such pathways must be in the observed set. We expect pathway enrichment to return more results than pathway covering when a larger number of metabolites are observed, resulting in more pathways that exceed the *p*-value cutoff. One of our motivations for developing the pathway covering method was that enrichment sometimes returns many “overlapping” pathways that are all triggered by a small number of metabolites that are found in multiple pathways.

Visualization approaches that center on interpreting metabolomics data focus on visualizing metabolites (either presence on a list or level of change between samples) on a diagram, either as a set of pathways or metabolic overview diagram. Examples include multiple tools in the Pathways Tools suite [18], also the PaintOmics [23] and MetaMapp [24] tools which integrate interpretation of spectra with metabolite visualization such as Pathos [25].

Pathway covering, as implemented on the BioCyc website, provides visualization of metabolites in pathways but is really an alternative to enrichment methods. It differs from enrichment methods in that it can propose alternative solutions, both because of alternative cost functions and because of the existence of multiple solutions with the same objective function value. Enrichment approaches, for example, simply count compounds and pathways, whereas cost functions can focus on properties of pathways in addition to whether the pathway contain a particular compound as a substrate (as exemplified by our pathway harmony function).

Pathway covering proposes a small set of pathways, which potentially makes a solution easier for the user to evaluate than either a large set of enriched pathways, or a graphical display of compounds mapped onto a large and complex metabolic map diagram.

### 3.1. Comparing Cost Functions

We evaluated five cost functions. Although in many cases an input compound will be mapped to a particular reaction regardless of the cost function, the functions did produce different results. The most important difference was the number of alternative solutions generated by a particular cost function. Although it is desirable to produce small, dense covering sets that reflect a strong signal in the data, a cost function that results in a multitude of solutions will also hide any such signal.

Although the constant cost function is the simplest method and will produce the smallest solution sets, it suffers from being the most prone to multiple solutions and will, more than any other cost function, choose large superpathways over smaller sets of pathways that cover the same compounds. The pathway size and covered compound sparseness functions avoid these issues, but by using the proportion of covered input compounds to other substrates, covered compound sparseness makes better use of the provided information and seems to be intermediate between pathway size and constant cost in its selections. It was also the best at returning single or very small sets of solutions. The biosynthesis-preferred function did show the preference intended, but otherwise behaves like pathway size. Finally, pathway harmony is the only cost function that does distinguish between increasing and decreasing compounds in the input list, and when it detects a pattern in a pathway’s reactants or products, it does produce unique pathway choices. However, when the majority of pathways cover only a small number of compounds, pathway harmony acts very much like the constant weight function, and leads to the same problem of large numbers of alternative solutions. The use of pathway harmony is strongly discouraged for data sets where increases and decreases in compound levels are not specified, since behavior resembling the constant cost function is almost guaranteed.

Unless there is a specific need for a focus on a focus on synthesis, we recommend the Covered Compound Sparseness or Pathway Harmony (when appropriate) cost functions.

### 3.2. Software Use and Availability

The Pathway Covering tool is available as part of BioCyc.org and the Pathway Tools software, version 23.0 and higher, under the web Analysis menu. (Note to reviewers: The Pathway Covering tool will be released in the next BioCyc.org release in spring 2019. But it is now available at our beta-test site at the following URL for testing by reviewers under the Analysis menu: brg-preview.ai.sri.com.) The user first selects the organism database that will supply the pathways for which the covering will be computed. The tool next reads a file of metabolites in a one-compound-per-line format, with an optional second column indicating whether the compound increased (“+”) or decreased (“−”). The tool enables the user to select among the cost functions described in here.

The tool will report which compounds were recognized by the selected organism database and which of those compounds have one or more pathways in the database (requirements for being included in the pathway covering). On a separate browser tab, the set of compounds in the covering solution will be shown, giving the pathway’s score and a small diagram of the pathway with each covered compound’s location in the pathway highlighted.

The Common Lisp source code, which includes additional APIs and cost functions, is available in the Supplemental Materials (S1). The file includes user entry points and the required execution environment (Pathway Tools 23.0 with SCIP linked into the image). We also include a sample input file in the Supplemental Materials (S2).

## 4. Materials and Methods

### 4.1. Selection and Processing of Datasets

We selected three human data sets, two from MWB and the other supplementary data to a publication.

- “Metabolic Profiling of Visceral and Subcutaneous Adipose Tissue from Colorectal Cancer Patients: GC-TOF MS Analysis of Subcutaneous and Visceral Adipose Tissue Samples,” which was Metabolomics Workbench Study ST000061.
- “Metabolite-Phenotype Link in X-linked Adrenoleukodystrophy (Fibroblast Cell Culture),” which was Metabolomics Workbench Study ST000741.
- “McDonnell et al.” used supplementary data from [11]

Although the software will work with any of the more than 14,000 PGDBs available in BioCyc, we focused on human metabolomics data because of the large number of human studies and availability of HumanCyc, a highly curated PGDB. The selection process focused on studies that had sizable numbers of non-lipid metabolites that showed significant and sizable differences between groups. The volcano plot and ANOVA tools in Metabolomics Workbench were used for the initial screening; we used (Benjamini-Hochberg corrected) *p*-values of 0.05 and 1.5-fold abundance differences in the screening.

For the McDonnell et al. study, we used the list of metabolites and the same 1.5-fold abundance difference for screening.

Study ST000741 and the McDonnell list yielded lists of metabolites that increased or decreased. The implementation supports entering the list of increased and decreased metabolites separately, but the lists are combined internally except when the pathway harmony cost function is used. All the differences in study ST000061 were in one direction.

### 4.2. Cost Functions

We consider five cost functions in this study. They are summarized here:

- **Constant**—This function returns a constant cost for every pathway. This cost function corresponds to treating pathway covering as a special case of set covering; all pathways are treated equally.
  (1) cost=1
- **Pathway Size**—This function returns the number of reactions in the pathway as its cost, so that small pathways are preferred to large pathways.
  (2) cost=|reactionsOfPathway|
- **Biosynthesis Preference**—This function starts with the pathway size cost, then, unless the pathway is a biosynthetic pathway, as determined by its location in the MetaCyc pathway ontology, the cost is multiplied by two and one is added.
  $$cost=\left\{\begin{array}{cc} 2|\mathrm{reactionsOfPathway}|+1 & \mathrm{when}\;\mathrm{pathway}\;\mathrm{is}\;\mathrm{biosynthetic}\\ \left|\mathrm{reactionsOfPathway}\right| & \mathrm{otherwise} \end{array}\right.$$
- **Covered Compound Sparseness**—This function prefers pathways that contain a large fraction of the input metabolites. The function computes a set *S*, the substrate compounds for all reactions in the pathway. It divides *S* into two subsets:
  1. SI—those compounds that are in the set of input compounds (*M*), which we call SI, and
  2. *R*—the remaining substrates.
  The multiplication and rounding assures an integer coefficient for the solver.
  (3) cost=round(1000|R|÷|SI|)
- **Pathway Harmony**—This function favors pathways where either the “input” substrates are metabolites that show an increase in abundance, and the “output” substrates are metabolites that show a decrease, or vice versa. This is the only cost function that distinguishes metabolites that increase or decrease in abundance. It divides the substrate compounds in the pathway into three groups:
  - compounds that are reactants, but not products of any reaction in the pathway (*R*),
  - compounds that are products, but not reactants of any reaction in the pathway (*P*), and
  - intermediates (neither reactants nor products, *I*)
  It then forms six intersections:
  - compounds that are increased in the treatment and are only reactants in the pathway (R+)
  - compounds that are decreased in the treatment and are only reactants in the pathway (R−)
  - compounds that are increased in the treatment and are only products in the pathway (P+)
  - compounds that are decreased in the treatment and are only products in the pathway (P−)
  - compounds that are increased in the treatment and are intermediates (I+)
  - compounds that are decreased in the treatment and are intermediates (I−)
  Pathway harmony then looks at the size of each intersection and compares the number of increasing and decreasing compounds within the three groups. It then assigns a lower (better score) when all compounds agree in direction within a group. Cases where most compounds in a group are in the same direction (e.g., one covered input compound increased while two decreased) are given a smaller cost advantage. For more details, refer to the calculate-harmony function in the source-code supplement (S1).
  $$cost=\left\{\begin{array}{cc} 1 & \mathrm{if}\;agree(R+,R-)\;and\;agree(P+,P-)\;and\;agree(I+,I-)\\ 2 & \mathrm{if}\;agree(R+,R-)\;and\;agree(P+,P-)\\ 3 & \mathrm{if}\;(agree(R+,R-)\;and\;agree(I+,I-))\;or\;(agree(P+,P-)\;and\;agree(I+,I-))\\ 4 & \mathrm{if}\;majority(R+,R-)\;and\;majority(P+,P-)\;and\;majority(I+,I-)\\ 5 & \mathrm{if}\;majority(R+,R-)\;and\;majority(P+,P-)\;\\ 6 & \mathrm{if}\;(R\;\neq \emptyset \;or\;P\;\neq \emptyset )\;and\;((majority(R+,R-)\;and\;majority(I+,I-))\;or\;(majority(P+,P-)\;and\;majority(I+,I-)))\\ 7 & \mathrm{if}\;R\;\neq \emptyset \;and\;P\;\neq \emptyset \;and\;(majority(R+,R-)\;or\;majority(P+,P-))\\ 8 & \mathrm{if}\;agree(I+,I-)\\ 9 & \mathrm{if}\;I+\;\neq \emptyset \;and\;I-\;\neq \emptyset \;and\;majority(I+,I-)\\ 10 & otherwise \end{array}\right.$$
  where
  $$\begin{array}{c} agree(a,b)=(|a|\;>\;1\;and\;b=\emptyset )\;or\;(|b|>\;1\;and\;a=\emptyset )\\ majority(a,b)=(|a|\;>|b|\;and\;|a|\;>\;1)\;or\;(|b|\;>\;|a|\;and\;|b|\;>\;1) \end{array}$$
  Pathway harmony focuses on agreement within each of the three classes of substrates as indication that the experimental change or disease condition is affecting the pathway in a consistent way. A high flux through the pathway should increase the levels of all three classes, but if the pathway represents a bottleneck, the direction of change between the substrates might differ.

### 4.3. Computing a Pathway Covering

Computing a pathway covering is a process of five or six steps, depending on whether multiple solutions are desired.

(1) Select the BioCyc PGDB for the organism in which the metabolomics study was performed.
(2) Identify pathways in that PGDB that contain at least one substrate compound from the input set. These are subsequently referred to as candidate pathways. Other pathways (those with no substrate compounds in the input set) are ignored, since they would not be in the solution.
(3) Calculate the cost of each candidate pathway using the user-selected cost function.
(4) Use the costs and the compounds associated with each pathway to construct an integer programming problem.
(5) Solve the integer programming problem for the optimum value of the objective function
(6) If multiple solutions are desired, add the optimum value as a constraint to the integer programming problem and run the solver in multiple solution mode.

We use the SCIP solver [26,27], version 6.0. The SCIP solver can, under some circumstances, find multiple solutions for a problem. These solutions will have the same minimum value of the objective function, and we found that these solutions only differed by substitution of single pathways. Because the solver would not always generate all solutions, we did not investigate this thoroughly.

The integer programming problem is specified as an objective function to minimize, and a set of constraining inequalities. The objective function is created from all input pathways, summing the cost of each pathway in the solution set:

(4) $$\mathrm{Minimize}\sum_{i\in \mathrm{pathways}}p_{i}w_{i}$$

where each $p_{i}$ is a binary variable that is 1 if pathway *i* is in a solution and $w_{i}$ is the calculated cost of pathway *i*.

The reminder of the problem consists of constraints, expressed as inequalities, one per compound in the input set, to ensure that each compound is covered by at least one pathway in the solution given:

(5) $$c_{j}:\;\sum_{p_{i}\;covers\;c_{j}}p_{i}\geq 1$$

where the $p_{i}$ are binary variables as in the objective function.

For example, if compound *A* is covered only by pathways 1, and 2 and compound *B* is covered only by pathways 2 and 3 then the constraints are:

(6) $$\begin{array}{c} c_{A}:\;P_{1}+P_{2}\geq 1\\ c_{B}:\;P_{2}+P_{3}\geq 1 \end{array}$$

All such constraints can be satisfied, because the preprocessing of the input metabolites and pathways has ensured that each input metabolite is covered by at least one pathway.

These constraint inequalities and the objective function are passed to the solver for the case of solving for a single solution. The solver will return both a solution and the corresponding (optimum) value for the objective function. If multiple solutions are requested, an additional constraint is added where the value of the objective function is less than one more than the previously returned optimum value (this relaxation of the constraint allows for rounding errors as well as the possibility of near optimal solutions).

A worked-through example appears in Appendix B.

### 4.4. HumanCyc Pathways Not Included in the Analysis

The covering algorithm ignores non-metabolic pathways such as transport and specifically signaling pathways, and the “pathway” comprised of the amino acid charging reactions for each tRNA.

### 4.5. Comparison with Random Metabolite Sets

We generated random sets of metabolites by gathering all the substrates of reactions within pathways (some reactions are not part of pathways in HumanCyc) into a single list. For each study, a list of 1000 sets of random metabolites of size equal to the number of metabolites for each study was generated. The metabolites on these lists were limited to those found in pathways. As the purpose of this analysis was determining that the pathway covering was detecting some signal in the metabolite input, the simplest and most compact cost function (constant) was used for the comparison of each study and generation of the distribution of pathway sizes.

### 4.6. Comparison with Enrichment Analysis

We performed pathway enrichment analysis on each of the three datasets. We used BioCyc’s SmartTables enrichment analysis to determine pathways enriched for each set, using HumanCyc as the selected database. We specified a Benjamini-Hochberg correction for multiple comparisons and a *p*-value cutoff of 0.1 as representing typical settings one would use in an exploratory analysis. Please note that for study ST000741, as noted in the results, applying any correction resulted in no enriched pathways with a *p*-value less than 0.3, so the set of pathways reported are those with no correction applied.

## Figures and Tables

**Table 1:** Selection and filtering of compounds.

Study	Significant Compounds in MWB	Recognized Compounds	Recognized Compounds in Pathways
ST000061	39	42 ^1^	35
ST000741	25 (12 increased)	25 ^2^	20
McDonnell et al.	24 (6 higher in control)	23	19

${}^{1}$ Compound glyceric acid was resolved into four related compounds. ${}^{2}$ Two compounds were not found in HumanCyc, but “fructose” and “glucose” were each resolved into two related compounds.

**Table 2:** ST000061 metabolites higher in visceral tissue than subcutaneous tissue.

arachidonic acid	aspartic acid	methionine
malic acid	lysine	phenylalanine
uracil	alanine	glyceric acid ^1^
threonine	serine	oxoproline
glutamate	histidine	xanthine
ornithine	tyrosine	leucine
glycerol	cholesterol	proline
isoleucine	fumaric acid	putrescine
hypoxanthine	valine	ethanolamine
citric acid	guanosine	inosine
tryptophan	alpha-tocopherol	

${}^{1}$ Compound glyceric acid was resolved into 3-phospho-d-glycerate, 2,3-diphospho-d-glycerate, 3-phospho-d-glceroyl-phosphate, and 2-phospho-d-glycerate.

**Table 3:** Summary of ST000061 Coverings by Cost Function.

Cost Function	Number of Solutions	Solution Size	Largest Covering
Constant	>$10^{6}$	20	7
Pathway size	324	23–24	4
Biosynthesis-preferred	162	24	4
Covered compound sparseness	4	21–22	7

**Table 4:** Comparison of ST000061 Pathway Enrichment to Pathway Covering using Covered Compound Sparseness.

Compound	Enrichment	Covering-Sparseness
adenosine nucleotides degradation	X	
L-alanine biosynthesis	X	
L-alanine degradation	X	
L-tyrosine degradation	X	
anandamide degradation	X	X
L-aspartate biosynthesis	X	X
purine nucleotides degradation	X	
purine ribonucleosides degradation to ribose-1-phosphate	X	
superpathway of purine nucleotide salvage	X	
alpha-tocopherol degradation		X
gamma-glutamyl cycle		X
5-oxo-l-proline metabolism		X
glycine betaine degradation II (mammalian)		X
histamine biosynthesis		X
L-isoleucine degradation		X
L-tyrosine biosynthesis		X
L-leucine degradation		X
L-lysine degradation I (saccharopine pathway)		X
phosphatidylserine biosynthesis II		X
pregnenolone biosynthesis		X
proline degradation		X
purine ribonucleosides degradation to ribose-1-phosphate		X
putrescine biosynthesis I		X
pyrimidine ribonucleosides degradation		X
S-adenosyl-l-methionine biosynthesis		X
superpathway of conversion of glucose to acetyl CoA and entry into the TCA cycle		X
threonine degradation		X
triacylglycerol degradation		X
tryptophan degradation to 2-amino-3-carboxymuconate semialdehyde		X
valine degradation		X

**Table 5:** ST000741, Metabolites increased in cultures from patients with severe disease.

Increased in Disease	Decreased in Disease
arachidonic acid	cis-aconitate
choline	phosphocreatine
*n*-acetylneuraminate	fumarate
taurine	alanine
fructose	5-methylthioadenosine
glucose	sn-glycero-3-phosphocholine
myo-inositol	nicotinamide
docosahexaenoic acid	urate
	malate
	ophthalmic acid

**Table 6:** Summary of ST00741 Coverings by Cost Function.

Cost Function	Number of Solutions	Solution Size	Largest Covering
Constant	6720	14	3
Pathway size	1	14	3
Biosynthesis-preferred	12	14	3
Covered compound sparseness	1	14	3
Pathway harmony	6720	14	3

**Table 7:** Comparison of ST000741 Pathway Enrichment to Pathway Covering using Covered Compound Sparseness.

Compound	Enrichment	Covering-Sparseness
adenosine ribonucleotides de novo biosynthesis	X	
anandamide degradation	X	X
aspirin-triggered lipoxin biosynthesis	X	
C20 prostanoid biosynthesis	X	
choline degradation	X	
creatine-phosphate biosynthesis	X	X
D-myo-inositol (1,4,5)-trisphosphate degradation	X	
glycine biosynthesis	X	
guanosine nucleotides degradation	X	
L-alanine biosynthesis	X	
L-alanine degradation	X	
15-eps-lipoxin biosynthesis	X	
myo-inositol de novo biosynthesis	X	X
NAD salvage	X	X
ophthalmate biosynthesis	X	X
phosphatidylcholine biosynthesis	X	
phosphatidylserine biosynthesis I	X	
phospholipases	X	
plasmalogen degradation	X	X
S-methyl-5’-thioadenosine degradation	X	X
spermidine biosynthesis	X	
spermine biosynthesis	X	
superpathway of conversion of glucose to acetyl CoA and entry into the TCA cycle	X	
superpathway of D-myo-inositol (1,4,5)-trisphosphate metabolism	X	
taurine biosynthesis	X	
TCA cycle	X	X
thio-molybdenum cofactor biosynthesis	X	
tRNA-uridine 2-thiolation (mammalian mitochondria)	X	X
urate biosynthesis/inosine 5’-phosphate degradation	X	X
biosynthesis/inosine 5’-phosphate degradation	X	
aspirin triggered resolvin D biosynthesis		X
CMP-*N*-acetylneuraminate biosynthesis I (eukaryotes)		X
sorbitol degradation I		X
trehalose degradation		X

**Table 8:** McDonnell 2013, metabolite set.

Higher in Control	Higher in Treated
uridine	uracil
cytidine	5-phospho-alpha-d-ribose 1-diphosphate
deoxycytidine	orotate
guanine	S-dihydroorotate
2-amino-2-deoxy-d-gluconate	dTMP
sn-glycero-3-phosphocholine	cytosine
	5,6-dihydrouracil
	2’3’-Cyclic CMP
	AMP
	D-Ribose 5-phosphate
	glycerone phosphate
	S-lactate
	NAD+
	succinate
	hexadecanoic acid
	tetradecanoic acid
	CoA
	glutarate

**Table 9:** Summary of McDonnell 2013 Coverings by Cost.

Cost Function	Number of Solutions	Solution Size	Largest Covering
Constant	7	8	5
Pathway size	4	11–12	3
Biosynthesis-preferred	1	11	3
Compound sparseness	1	10	4
Pathway harmony	64	8–9	4

**Table 10:** Comparison of McDonnell Pathway Enrichment to Pathway Covering using Covered Compound Sparseness.

Compound	Enrichment	Covering-Sparseness
2-oxoglutarate decarboxylation to succinyl-CoA	X	
2-oxoisovalerate decarboxylation to isobutanoyl-CoA	X	
2’-deoxy-alpha-d-ribose 1-phosphate degradation	X	
4-aminobutyrate degradation	X	
acetate conversion to acetyl CoA	X	
adenine and adenosine salvage I	X	
arachidonate biosynthesis III (metazoa)	X	
beta-alanine degradation	X	
coenzyme A biosynthesis II (eukaryotic)	X	
ethanol degradation II	X	
ethanol degradation III	X	
ethanol degradation IV	X	
fatty acid alpha-oxidation	X	
fatty acid alpha-oxidation III	X	
fatty acid beta-oxidation	X	
fatty acid beta-oxidation (peroxisome)	X	
fatty acid activation	X	
glycerol-3-phosphate shuttle	X	X
guanine and guanosine salvage	X	X
ketolysis	X	X
lactate fermentation (reoxidation of cytosolic NADH)	X	
long-chain fatty acid activation	X	
L-threonine degradation	X	
NAD de novo biosynthesis	X	
NAD biosynthesis from 2-amino-3-carboxymuconate semialdehyde	X	
NAD salvage	X	
phytol degradation	X	
PRPP biosynthesis	X	X
purine nucleotides degradation	X	
pyrimidine deoxyribonucleosides degradation	X	X
pyrimidine ribonucleosides degradation	X	X
pyrimidine ribonucleosides salvage I	X	
pyruvate fermentation to (S)-lactate	X	X
sphingosine and sphingosine-1-phosphate metabolism	X	
stearate biosynthesis	X	X
superpathway of conversion of glucose to acetyl CoA and entry into the TCA cycle	X	
superpathway of purine nucleotide salvage	X	
superpathway of pyrimidine deoxyribonucleotides de novo biosynthesis	X	X
superpathway of pyrimidine ribonucleosides degradation		X
superpathway of pyrimidine ribonucleotides de novo biosynthesis	X	
TCA cycle	X	
UMP biosynthesis	X	
uracil degradation	X	
y-linolenate biosynthesis	X	
plasmalogen degradation		X

## Supplementary Materials

The following are available online at https://www.mdpi.com/2218-1989/9/5/88/s1. Common Lisp source code for the Pathway Covering algorithm This file contains the source code for the Pathway Covering Tool. It includes all the cost functions described in this paper as well as several additional functions that weinvestigated and decided not to persue further.

## Abbreviations

The following abbreviations are used in this manuscript:

PGDB	Pathway Genome Database
MWB	Metabolomics Workbench
MDPI	Multidisciplinary Digital Publishing Institute

## Appendix A. Detailed Results

This appendix provides details of the 14 pathway covering analyses described in the results. Each table lists the compounds shared by all solutions returned by the solver. Text below the table describes the differences in pathways among the solutions. The summary extracts several common patterns in the results: two pathways that alternate in different solutions (never in the same solution, but always one present); three pathways that alternate in different solutions; two pathways that always co-occur, usually alternating with a third pathway that covers the same compounds as the co-occurring pathways. Not all solutions can be completely broken down this way; in that case, the collection of pathways appearing in the remaining variation among solutions are simply listed.

### Appendix A.1. Results from MWB Study ST000061

This section includes results from four cost functions: constant (Table A1); pathway size (Table A2); biosynthesis preferred (Table A3); and covered compound sparseness (Table A4). The results for constant are the complete set of pathways for one solution. The results for pathway size, biosynthesis-preferred, and covered compound sparseness list the pathways found in all solutions and summarizes the variation among solutions. Pathway harmony is not included because all significant compound changes were in one direction, whereas pathway harmony is designed to respond to differences in metabolite change direction.

**Table A1:** ST000061, Representative Pathway Covering Using Constant Cost Function.

Pathway	Metabolites Covered
5-oxo-l-proline metabolism	5-oxo-l-proline, L-glutamate
alpha-tocopherol degradation	alpha-tocopherol
anandamide degradation	arachidonic acid, ethanolamine
histidine degradation	L-glutamate, L-histidine
isoleucine degradation	L-isoleucine, L-glutamate
leucine degradation	L-leucine, L-glutamate
lysine degradation I (saccharopine pathway)	L-glutamate, L-lysine
methionine salvage cycle III	L-methionine, putrescine
NAD de novo biosynthesis	L-tryptophan, L-alanine, L-glutamate
phenylalanine degradation/tyrosine biosynthesis	L-phenylalanine, L-tyrosine
pregnenolone biosynthesis	cholesterol
proline degradation	L-proline, L-glutamate
purine nucleotides degradation	xanthine, guanosine, hypoxanthine, inosine
pyrimidine deoxyribonucleosides degradation	uracil
superpathway of conversion of glucose to acetyl CoA and entry into the TCA cycle	2-phospho-d-glycerate, 3-phospho-d-glycerate,
	3-phospho-d-glyceroyl-phosphate, (S)-malate,
	fumarate, citrate, 2,3-diphospho-d-glycerate
superpathway of methionine degradation	L-methionine, L-glutamate, L-serine
threonine degradation	L-threonine
triacylglycerol degradation	glycerol
urea cycle	L-ornithine, fumarate, L-aspartate
valine degradation	L-glutamate, L-valine

Unlike the remaining appendix tables, Table A1 summarizes a single representative pathway covering solution out of over $10^{6}$ solutions possible for this cost function and this metabolite set.

In addition to the pathways listed in Table A2, there are two pairs of alternative pathways: fumarate is covered by adenosine ribonucleotides de novo biosynthesis or the citrulline-nitric oxide cycle; 5-oxo-l-proline is covered by either 5-oxo-l-proline metabolism or the gamma-glutamyl cycle.

Likewise, in addition to the pathways in Table A2, there are two sets of three-way alternative pathways: histidine is covered by either histamine biosynthesis, homocarnosine biosynthesis, or carnosine biosynthesis; L-serine is covered by either glycine/serine biosynthesis, phosphatidylserine biosynthesis I or phosphatidylserine biosynthesis II. The remaining compounds are covered by a combination of pathways drawn from the following set: L-alanine biosynthesis, L-alanine degradation, L-tryptophan degradation to 2-amino-3-carboxymuconate semialdehyde, L-tryptophan degradation via tryptamine, glycine biosynthesis, serotonin and melatonin biosynthesis, thio-molybdenum cofactor biosynthesis.

In addition to the pathways listed in Table A3, there is one pair of alternative pathways: fumarate is covered by either adenosine ribonucleotides de novo biosynthesis or the citrulline-nitric oxide cycle.

Likewise, in addition to the pathways in Table A3, there are two pairs of pathways that always appear together in a covering solution. The pathways guanine and guanosine salvage and adenosine nucleotides degradation appear together and cover guanosine, inosine, hypoxanthine, and xanthine. The pathways adenine and adenosine salvage III and guanosine nucleotides degradation appear together and cover guanosine, inosine, hypoxanthine, and xanthine.

There are four sets of three-way alternative pathways in addition to the pathways listed in Table A3 and the preceding groups and alternative pathways. The compounds and their covering pathway alternatives are histidine is covered by either histamine biosynthesis, homocarnosine biosynthesis, or carnosine biosynthesis; L-serine is covered by either glycine/serine biosynthesis, phosphatidylserine biosynthesis I or phosphatidylserine biosynthesis II; guanosine, inosine, hypoxanthine and xanthine are covered by either adenine and adenosine salvage III, guanine and guanosine salvage, or purine ribonucleosides degradation to ribose-1-phosphate; L-alanine is covered by thio-molybdenum cofactor biosynthesis, glycine biosynthesis, or L-alanine biosynthesis.

In addition to the pathways listed in Table A4, The pair of pathways S-adenosyl-l-methionine biosynthesis and phosphatidylserine biosynthesis II always appear together in a covering solution and together cover L-serine, ethanolamine, and L-methionine.

Also, in addition to the pathways listed in Table A4 the preceding occurring pair alternates with glycine betaine degradation II (mammalian). The compound 5-oxo-l-proline is covered by either the gamma-glutamyl cycle or 5-oxo-l-proline metabolism.

**Table A2:** ST000061, Pathways Occurring in All Solutions Using Pathway Size Cost Function.

Pathway	Metabolites Covered
alpha-tocopherol degradation	alpha-tocopherol
acetyl CoA biosynthesis from citrate	citrate
anandamide degradation	arachidonic acid, ethanolamine
glycerol degradation	glycerol
L-isoleucine degradation	L-isoleucine, L-glutamate
L-leucine degradation	L-leucine, L-glutamate
L-lysine degradation I (saccharopine pathway)	L-glutamate, L-lysine
malate-aspartate shuttle	(S)-malate, L-aspartate, L-glutamate
tyrosine biosynthesis	L-phenylalanine, L-tyrosine
pregnenolone biosynthesis	cholesterol
proline degradation	L-proline, L-glutamate
purine ribonucleosides degradation to ribose-1-phosphate	guanosine, inosine, hypoxanthine, xanthine
putrescine biosynthesis III	L-ornithine, putrescine
pyrimidine ribonucleosides degradation	uracil
Rapoport-Luebering glycolytic shunt	2,3-diphospho-d-glycerate, 3-phospho-d-glycerate,
	3-phospho-d-glyceroyl-phosphate, 2-phospho-d-glycerate
S-adenosyl-l-methionine biosynthesis	L-methionine
threonine degradation	L-threonine
valine degradation	L-glutamate, L-valine

**Table A3:** ST000061, Pathway Covering Using Biosynthesis-Preferred Cost Function.

Pathway	Metabolites Covered
acetyl CoA biosynthesis from citrate	citrate
alpha-tocopherol degradation	alpha-tocopherol
anandamide degradation	arachidonic acid, ethanolamine
gamma-glutamyl cycle	5-oxo-l-proline, L-glutamate
glycerol degradation	glycerol
L-isoleucine degradation	L-isoleucine, L-glutamate
L-leucine degradation	L-leucine, L-glutamate
L-lysine degradation I (saccharopine pathway)	L-glutamate, L-lysine
L-proline biosynthesis	L-glutamate, L-proline
L-threonine degradation	L-threonine
L-tyrosine biosynthesis	L-phenylalanine, L-tyrosine
L-valine degradation	L-glutamate, L-valine
malate-aspartate shuttle	(S)-malate, L-aspartate, L-glutamate
pregnenolone biosynthesis	cholesterol
putrescine biosynthesis III	L-ornithine, putrescine
Rapoport-Luebering glycolytic shunt	2,3-diphospho-d-glycerate, 3-phospho-d-glycerate, 3-phospho-d-glyceroyl-phosphate, 2-phospho-d-glycerate
S-adenosyl-l-methionine biosynthesis	L-methionine
serotonin and melatonin biosynthesis	L-tryptophan
thio-molybdenum cofactor biosynthesis	L-alanine
uracil degradation I	uracil

**Table A4:** ST000061, Pathway Covering Using Covered Compound Sparseness Cost Function.

Pathway	Metabolites Covered
alpha-tocopherol degradation	alpha-tocopherol
anandamide degradation	arachidonic acid, ethanolamine
histamine biosynthesis	L-histidine
L-aspartate biosynthesis	L-aspartate, L-glutamate
L-isoleucine degradation	L-isoleucine, L-glutamate
L-leucine degradation	L-leucine, L-glutamate
L-lysine degradation I (saccharopine pathway)	L-glutamate, L-lysine
L-proline degradation	L-proline, L-glutamate
L-threonine degradation	L-threonine
L-tryptophan degradation to 2-amino-3-carboxymuconate semialdehyde	L-tryptophan, L-alanine
L-tyrosine biosynthesis	L-phenylalanine, L-tyrosine
L-valine degradation	L-glutamate, L-valine
pregnenolone biosynthesis	cholesterol
purine ribonucleosides degradation to ribose-1-phosphate	guanosine, inosine, hypoxanthine, xanthine
putrescine biosynthesis III	L-ornithine, putrescine
pyrimidine ribonucleosides degradation	uracil
superpathway of conversion of glucose to acetyl CoA and entry into the TCA cycle	2-phospho-d-glycerate, 3-phospho-d-glycerate,
	3-phospho-d-glyceroyl-phosphate, (S)-malate, fumarate,
	citrate, 2,3-diphospho-d-glycerate
triacylglycerol degradation	glycerol

### Appendix A.2. Results from MWB Study ST000741

This section includes results of five cost functions: constant (Table A5); pathway size (Table A6); biosynthesis-preferred (Table A7); sparseness (Table A8); and pathway harmony (Table A9).

In addition to the pathways listed in Table A5, there are three pairs of alternative pathways: fumarate is covered by either the TCA cycle or the superpathway of conversion of glucose to acetyl CoA and entry into the TCA cycle; beta-d-glucopyranose and alpha-d-glucopyranose are covered by either trehalose degradation or protein i>-glycosylation processing phase (mammalian); (4Z,7Z,10Z,13Z,16Z,19Z)-docosahexaenoate is covered either by aspirin triggered resolvin D biosynthesis, or by resolvin D biosynthesis.

The remaining compounds not mentioned in Table A5, are covered by a combination of pathways drawn from the following set: 15-epi-lipoxin biosynthesis, 3-phosphoinositide biosynthesis, 7-(3-amino-3-carboxypropyl)-wyosine biosynthesis, S-methyl-5’-thioadenosine degradation, myo-inositol biosynthesis, C20 prostanoid biosynthesis, D-myo-inositol (1,4,5)-trisphosphate biosynthesis, D-myo-inositol (1,4,5)-trisphosphate degradation, L-methionine salvage cycle, adenosine nucleotides degradation, anandamide degradation, diphthamide biosynthesis, guanosine nucleotides degradation, inosine 5’-phosphate degradation, leukotriene biosynthesis, lipoxin biosynthesis, purine nucleotides degradation, spermidine biosynthesis, spermine biosynthesis, superpathway of D-myo-inositol (1,4,5)-trisphosphate metabolism, superpathway of inositol phosphate compounds, wybutosine biosynthesis.

Table A6 lists the single solution for the pathway size cost function for ST000741.

In addition to the pathways listed in Table A7, there are two pairs of alternative pathways: 5-methylthioadenosine is covered by either spermine biosynthesis or spermidine biosynthesis; urate is covered by either guanosine nucleotides degradation or inosine 5’-phosphate degradation. There is also one set of three alternative pathways that cover L-alanine and includes thio-molybdenum cofactor biosynthesis, glycine biosynthesis, and L-alanine biosynthesis.

Table A8 lists the single solution for the covered compound sparseness cost function for ST000741.

In addition to the pathways listed in Table A9, there are three pairs of alternative pathways: fumarate, (S)-malate, and cis-aconitate are covered by either the TCA cycle or the superpathway of conversion of glucose to acetyl CoA and entry into the TCA cycle; (4Z,7Z,10Z,13Z,16Z,19Z)- docosahexaenoate is covered by either aspirin triggered resolvin D biosynthesis or resolvin D biosynthesis; alpha-d-glucopyranose and beta-d-glucopyranose are covered by either trehalose degradation or protein N-glycosylation processing phase (mammalian).

The remaining compounds not covered in Table A9, are covered by a combination of pathways drawn from the following set: 15-epi-lipoxin biosynthesis, 3-phosphoinositide biosynthesis, 7-(3-amino- 3-carboxypropyl)-wyosine biosynthesis, S-methyl-5’-thioadenosine degradation, myo-inositol biosynthesis, C20 prostanoid biosynthesis, D-myo-inositol (1,4,5)-trisphosphate biosynthesis, D-myo-inositol (1,4,5)-trisphosphate degradation, L-methionine salvage cycle, adenosine nucleotides degradation, anandamide degradation, diphthamide biosynthesis, guanosine nucleotides degradation, inosine 5’-phosphate degradation, leukotriene biosynthesis, lipoxin biosynthesis, purine nucleotides degradation, spermidine biosynthesis, spermine biosynthesis, superpathway of D-myo-inositol (1,4,5)-trisphosphate metabolism, superpathway of inositol phosphate compounds, wybutosine biosynthesis.

**Table A5:** ST000741, Pathway Covering Using Constant Cost Function.

Pathway	Metabolites Covered
CMP-*N*-acetylneuraminate biosynthesis I (eukaryotes)	*N*-acetyl-beta-neuraminate
creatine-phosphate biosynthesis	Nomega-phosphocreatine
NAD salvage	nicotinamide
ophthalmate biosynthesis	ophthalmate
plasmalogen degradation	choline, sn-glycero-3-phosphocholine
sorbitol degradation I	beta-d-fructofuranose, keto-d-fructose
tRNA-uridine 2-thiolation (mammalian mitochondria)	L-alanine, taurine

**Table A6:** ST000741, Pathway Covering Using Pathway Size Cost Function.

Pathway	Metabolites Covered
anandamide degradation	arachidonic acid
aspirin triggered resolvin D biosynthesis	(4Z,7Z,10Z,13Z,16Z,19Z)-docosahexaenoate
CMP-*N*-acetylneuraminate biosynthesis I (eukaryotes)	*N*-acetyl-beta-neuraminate
creatine-phosphate biosynthesis	Nomega-phosphocreatine
guanosine nucleotides degradation	urate
myo-inositol biosynthesis	myo-inositol
NAD salvage	nicotinamide
ophthalmate biosynthesis	ophthalmate
plasmalogen degradation	choline, sn-glycero-3-phosphocholine
S-methyl-5’-thioadenosine degradation	S-methyl-5’-thioadenosine
sorbitol degradation I	beta-d-fructofuranose, keto-d-fructose
TCA cycle	(S)-malate, fumarate, cis-aconitate
trehalose degradation	beta-d-glucopyranose, alpha-d-glucopyranose
tRNA-uridine 2-thiolation (mammalian mitochondria)	L-alanine, taurine

**Table A7:** ST000741, Pathway Covering Using Biosynthesis-Preferred Cost Function.

Pathway	Metabolites Covered
anandamide degradation	arachidonic acid
aspirin triggered resolvin D biosynthesis	(4Z,7Z,10Z,13Z,16Z,19Z)-docosahexaenoate
CMP-*N*-acetylneuraminate biosynthesis I (eukaryotes)	*N*-acetyl-beta-neuraminate
creatine-phosphate biosynthesis	Nomega-phosphocreatine
myo-inositol biosynthesis	myo-inositol
NAD salvage	nicotinamide
ophthalmate biosynthesis	ophthalmate
plasmalogen degradation	choline, sn-glycero-3-phosphocholine
sorbitol degradation I	beta-d-fructofuranose, keto-d-fructose
taurine biosynthesis	taurine
TCA cycle	(S)-malate, fumarate, cis-aconitate
trehalose degradation	beta-d-glucopyranose, alpha-d-glucopyranose

**Table A8:** ST000741, Pathway Covering Using Covered Compound Sparseness Cost Function.

Pathway	Metabolites Covered
anandamide degradation	arachidonic acid
aspirin triggered resolvin D biosynthesis	(4Z,7Z,10Z,13Z,16Z,19Z)-docosahexaenoate
CMP-*N*-acetylneuraminate biosynthesis I (eukaryotes)	*N*-acetyl-beta-neuraminate
creatine-phosphate biosynthesis	Nomega-phosphocreatine
inosine 5’-phosphate degradation	urate
myo-inositol de novo biosynthesis	myo-inositol
NAD salvage	nicotinamide
ophthalmate biosynthesis	ophthalmate
plasmalogen degradation	choline, sn-glycero-3-phosphocholine
S-methyl-5’-thioadenosine degradation	S-methyl-5’-thioadenosine
sorbitol degradation I	beta-d-fructofuranose, keto-d-fructose
TCA cycle	(S)-malate, fumarate, cis-aconitate
trehalose degradation	beta-d-glucopyranose, alpha-d-glucopyranose
tRNA-uridine 2-thiolation (mammalian mitochondria)	L-alanine, taurine

### Appendix A.3. Results Using Supplemental Data from McDonnell 2013

This section includes results of five cost functions: constant (Table A10); pathway size (Table A11); biosynthesis-preferred (Table A12); sparseness (Table A13); and pathway harmony (Table A14).

In addition to the pathways listed in Table A10, the remaining compounds are covered by a combination of pathways drawn from the following set: pyrimidine deoxyribonucleosides degradation, pyrimidine deoxyribonucleosides salvage, superpathway of pyrimidine deoxyribonucleoside salvage, superpathway of pyrimidine deoxyribonucleotides de novo biosynthesis, superpathway of pyrimidine ribonucleotides de novo biosynthesis, UMP biosynthesis.

In addition to the pathways listed in Table A11, the pair of pathways “pyrimidine ribonucleosides degradation” and "uracil degradation I (reductive)" always occur together in solutions and together cover uridine, uracil, cytidine, and 5,6-dihydrouracil. There are also two pairs of alternative pathways: glycerone phosphate and NAD+ are covered by glycerol degradation and glycerol-3-phosphate shuttle; the superpathway of pyrimidine ribonucleosides degradation is an alternative to the pair pyrimidine ribonucleosides degradation and uracil degradation I (reductive) mentioned previously.

Table A12 lists the single solution for the biosynthesis-preferred cost function for the McDonnell et al., 2013 data set.

Table A13 lists the single solution for the covered compound sparseness cost function for the McDonnell et al., 2013 data set.

In addition to the pathways listed in Table A14, the remaining compounds are covered by a combination of pathways drawn from the following set: 4-aminobutanoate degradation I, CDP-diacylglycerol biosynthesis, GABA shunt, L-carnitine biosynthesis, UMP biosynthesis, fatty acid alpha-oxidation, glycolysis, ketolysis, plasmalogen biosynthesis, pyrimidine deoxyribonucleosides degradation, pyrimidine deoxyribonucleosides salvage, superpathway of conversion of glucose to acetyl CoA and entry into the TCA cycle, superpathway of pyrimidine deoxyribonucleotides de novo biosynthesis, superpathway of pyrimidine ribonucleotides de novo biosynthesis.

**Table A9:** ST000741, Pathway Covering Using Pathway Harmony Cost Function.

Pathway	Metabolites Covered
CMP-*N*-acetylneuraminate biosynthesis I (eukaryotes)	*N*-acetyl-beta-neuraminate
creatine-phosphate biosynthesis	Nomega-phosphocreatine
NAD salvage	nicotinamide
ophthalmate biosynthesis	ophthalmate
plasmalogen degradation	choline, sn-glycero-3-phosphocholine
sorbitol degradation I	beta-d-fructofuranose, keto-d-fructose
tRNA-uridine 2-thiolation (mammalian mitochondria)	L-alanine, taurine

**Table A10:** McDonnell 2013, Pathway Covering Using Constant Cost Function.

Pathway	Metabolites Covered
plasmalogen degradation	sn-glycero-3-phosphocholine
purine ribonucleosides degradation to ribose-1-phosphate	guanine, D-ribose 5-phosphate
pyruvate fermentation to (S)-lactate	(S)-lactate, NAD+
stearate biosynthesis	coenzyme A, palmitate, AMP
superpathway of conversion of glucose to acetyl CoA and entry into the TCA cycle	NAD+, glycerone phosphate, coenzyme A, succinate
superpathway of pyrimidine deoxyribonucleotides de novo biosynthesis	(S)-dihydroorotate, orotate, 5-phospho-alpha-d-ribose 1-diphosphate, dTMP
superpathway of pyrimidine ribonucleosides degradation	uridine, uracil, cytidine, 5,6-dihydrouracil

**Table A11:** McDonnell 2013, Pathway Covering Using Pathway Size Cost Function.

Pathway	Metabolites Covered
4-aminobutyrate degradation	NAD+, succinate
guanine and guanosine salvage	guanine, 5-phospho-alpha-d-ribose 1-diphosphate
plasmalogen degradation	sn-glycero-3-phosphocholine
PRPP biosynthesis	D-ribose 5-phosphate, 5-phospho-alpha-d-ribose 1-diphosphate, AMP
pyrimidine deoxyribonucleosides salvage	dTMP, 2’-deoxycytidine
pyruvate fermentation to (S)-lactate	(S)-lactate, NAD+
stearate biosynthesis	coenzyme A, palmitate, AMP
UMP biosynthesis	(S)-dihydroorotate, orotate, 5-phospho-alpha-d-ribose 1-diphosphate

**Table A12:** McDonnell 2013, Pathway Covering Using Biosynthesis-Preferred Cost Function.

Pathway	Metabolites Covered
CDP-diacylglycerol biosynthesis	glycerone phosphate, coenzyme A
guanine and guanosine salvage	guanine, 5-phospho-alpha-d-ribose 1-diphosphate
L-carnitine biosynthesis	succinate, NAD+
pyruvate fermentation to (S)-lactate	(S)-lactate, NAD+
plasmalogen degradation	sn-glycero-3-phosphocholine
PRPP biosynthesis	D-ribose 5-phosphate, 5-phospho-alpha-d-ribose 1-diphosphate, AMP
pyrimidine deoxyribonucleosides salvage	dTMP, 2’-deoxycytidine
pyrimidine ribonucleosides salvage I	cytidine, uridine
stearate biosynthesis	coenzyme A, palmitate, AMP
UMP biosynthesis	(S)-dihydroorotate, orotate, 5-phospho-alpha-d-ribose 1-diphosphate
uracil degradation	5,6-dihydrouracil, uracil

**Table A13:** McDonnel 2013, Pathway Covering Using Covered Compound Sparseness Cost Function.

Pathway	Metabolites Covered
glycerol-3-phosphate shuttle	glycerone phosphate, NAD+
guanine and guanosine salvage	guanine, 5-phospho-alpha-d-ribose 1-diphosphate
ketolysis	coenzyme A, succinate, NAD+
pyruvate fermentation to (S)-lactate	(S)-lactate, NAD+
plasmalogen degradation	sn-glycero-3-phosphocholine
PRPP biosynthesis	D-ribose 5-phosphate, 5-phospho-alpha-d-ribose 1-diphosphate, AMP
pyrimidine deoxyribonucleosides degradation	uracil, 2’-deoxycytidine
super pathway of pyrimidine ribonucleosides degradation	uridine, uracil, cytidine, 5,6-dihydrouracil
stearate biosynthesis	coenzyme A, palmitate, AMP
superpathway of pyrimidine deoxyribonucleotides de novo biosynthesis	(S)-dihydroorotate, orotate, 5-phospho-alpha-d-ribose 1-diphosphate, dTMP

**Table A14:** McDonnell 2013, Pathway Covering Using Pathway Harmony Cost Function.

Pathway	Metabolites Covered
plasmalogen degradation	sn-glycero-3-phosphocholine
purine ribonucleosides degradation to ribose-1-phosphate	guanine, D-ribose 5-phosphate
pyruvate fermentation to (S)-lactate	(S)-lactate, NAD+
stearate biosynthesis	coenzyme A, palmitate, AMP
superpathway of pyrimidine ribonucleosides degradation	uridine, uracil, cytidine, 5,6-dihydrouracil

## Appendix B. Example Computation

Here is an example, using a small set of metabolites and using the EcoCyc database. Steps here are annotated to the corresponding steps in the “Computing a Pathway Covering” subsection of the methods.

- Compounds: ALPHA-GLUCOSE, L-ALPHA-ALANINE, FORMALDEHYDE, VALINE
- Organism E. coli K12 MG1655
- Cost function: pathway size (number of reactions)

Selecting the EcoCyc database corresponds to the first step.

Of 432 pathways (excluding tRNA charging) in the organism, only 19 contain one or more of the input compounds. These are:

- biotin biosynthesis I [38 metabolites, 1 covered]
- superpathway of L-alanine biosynthesis [37 metabolite, 1 covered]
- UDP-*N*-acetylmuramoyl-pentapeptide biosynthesis I (*meso*-diaminopimelate containing) [20 metabolites, 1 covered]
- formaldehyde oxidation II (glutathione-dependent) [9 metabolites, 1 covered]
- 8-amino-7-oxononanoate biosynthesis I [24 metabolites, 1 covered]
- trehalose degradation VI (periplasmic) [4 metabolites, 1 covered]
- trehalose degradation II (cytosolic) [9 metabolites, 1 covered]
- thiazole biosynthesis I (facultative anaerobic bacteria) [29 metabolites, 1 covered]
- L-alanine biosynthesis I [7 metabolites, 2 covered]
- L-valine biosynthesis [12 metabolites, 1 covered]
- L-alanine degradation I [9 metabolites, 1 covered]
- L-alanine biosynthesis II [4 metabolites, 1 covered]
- L-alanine biosynthesis III [4 metabolites, 1 covered]
- molybdenum cofactor biosynthesis [24 metabolites, 1 covered]
- tRNA-uridine 2-thiolation (bacteria) [33 metabolites, 1 covered]
- superpathway of branched chain amino acid biosynthesis [31 metabolites, 1 covered]
- muropeptide degradation [9 metabolites, 1 covered]
- peptidoglycan biosynthesis I (*meso*-diaminopimelate containing) [25 metabolites, 1 covered]
- superpathway of thiamine diphosphate biosynthesis I [37 metabolites, 1 covered]

This corresponds to the second step in the Methods subsection.

The weights for each pathway are calculated using the selected cost function (pathway size), which is the third step in the Methods subsection.

The fourth step is constructing the problem. The objective function is the sum of a set of variables, one for each candidate pathway ($P_{1}$ through $P_{19}$ for this problem), where each pathway variable is multiplying by the weight of its pathway. The remaining constraints are constructed one for each compound in the input set, and are the sum of the variable for each pathway that covers the compound, constrained to be greater or equal to 1.

Minimize:

(A1) $$\begin{array}{c} 14P_{1}+5P_{2}+8P_{3}+3P_{4}+11P_{5}+2P_{6}+8P_{7}+8P_{8}+7P_{9}+4P_{10}+\\ 2P_{11}+2P_{12}+P_{13}+3P_{14}+P_{15}+17P_{16}+4P_{17}+10P_{18}+11P_{19} \end{array}$$

Subject to these constraints:

(A2) $$P_{11}+P_{12}\geq 1$$

(A3) $$P_{4}\geq 1$$

(A4) $$P_{19}+P_{6}+P_{7}+P_{8}+P_{9}+P_{1}+P_{10}+P_{2}+P_{13}+P_{14}+P_{15}+P_{3}+P_{18}+P_{5}\geq 1$$

(A5) $$P_{16}+P_{17}+P_{2}+P_{14}\geq 1$$

The fifth step is passing the problem to the solver, requesting a single solution. The solver returns a solution:

- formaldehyde oxidation II (glutathione-dependent); covers formaldehyde
- L-alanine biosynthesis I; covers valine and alanine
- trehalose degradation II (cystolic); covers alpha-glucose

It also returns the optimum value (8.0) corresponding to the cost for this solution (3+3+2).

If multiple solutions are requested, a sixth step is required. A constraint setting the objective function to be equal to the optimum value is added. In this example, a second solution with trehalose degradation VI (periplasmic) replacing trehalose degradation II is returned. The substitution does not affect the objective as both pathways have a reaction count of 2. Please note that finding the optimum value to use as a constraint appears to be a necessary approach when using SCIP as a solver. Other solvers may differ.
